# Psychometric properties of the Brazilian version of the Early Childhood Oral Health Impact Scale (B-ECOHIS)

**DOI:** 10.1186/1472-6831-11-19

**Published:** 2011-06-13

**Authors:** Ana Carolina Scarpelli, Branca Heloísa Oliveira, Flávia C Tesch, Anna Thereza Leão, Isabela A Pordeus, Saul M Paiva

**Affiliations:** 1 Department of Pediatric Dentistry and Orthodontics, Faculty of Dentistry, Federal University of Minas Gerais - Av. Antônio Carlos 6627, Belo Horizonte, MG, 31270-901, Brazil; 2 Department of Community and Preventive Dentistry, Faculty of Dentistry, Rio de Janeiro State University - Av. 28 de Setembro 157, Rio de Janeiro, RJ, 20551-030, Brazil; 3 Department of Dental Clinics, Federal University of Rio de Janeiro - Av. Brigadeiro Trompowsky, Rio de Janeiro, RJ, 21941-590, Brazil

## Abstract

**Background:**

Oral disorders can have a negative impact on the functional, social and psychological wellbeing of young children and their families and cause pain/discomfort for the child. Oral health-related quality of life (OHRQoL) has emerged as an important health outcome in clinical trials and healthcare research. The Early Childhood Oral Health Impact Scale (ECOHIS) is a proxy measure of children's OHRQoL designed to assess the negative impact of oral disorders on the quality of life of preschool children. The objective of this study was to evaluate the psychometric properties of the Brazilian version of the ECOHIS (B-ECOHIS).

**Methods:**

This investigation was carried out in preliminary and field studies. The preliminary study comprised a cross-sectional study carried out in the city of Petropolis, Brazil. A sample of 150 children from two to five years of age was recruited at a public hospital. In the field study, an epidemiological survey was carried out in public and private preschools of Belo Horizonte, Brazil. The B-ECOHIS was answered by 1643 parents/caregivers of five-year-old male and female preschool children. In both phases, oral examinations were performed by a single previously calibrated dentist. Reliability was determined through test-retest reliability and internal consistency. Validity was determined through convergent and discriminant validities. The correlation between the scores obtained on the child and family impact sections was assessed.

**Results:**

In the preliminary (P) and field (F) study, test-retest reliability correlation values were 0.98 and 0.99 for the child impact section and 0.97 and 0.99 for the family impact section, respectively. The B-ECOHIS demonstrated internal consistency: child impact section (P: α = 0.74; F: α = 0.80) and family impact section (P: α = 0.59; F: α = 0.76). The correlation between the scores obtained on the child and family impact sections was statistically significant (P: r_s _= 0.54; F: r_s _= 0.62; p ≤ 0.001). In both phases of the study, B-ECOHIS scores were significantly associated with the decayed, missing and filled teeth index, decayed teeth and discolored upper anterior teeth (p < 0.05).

**Conclusion:**

The B-ECOHIS proved reliable and valid for assessing the negative impact of oral disorders on the quality of life of preschool children.

## Background

Despite recent improvements in oral health, problems remain in many communities around the world particularly among underprivileged groups. Dental caries is the most prevalent oral disease in several Asian and Latin American countries, while it appears to be less common and less severe in most African countries. It is a major oral health problem in most industrialized countries, affecting 60-90% of schoolchildren [1]. Oral disorders can have a significant negative impact on the functional, social and psychological wellbeing of young children and their families [2]. This issue has resulted in a greater clinical focus on the measurement of quality of life as a complement to the assessment of oral health needs, the prioritization of care and evaluating the outcomes of treatment strategies [3].

Over the past decades, oral health-related quality of life assessment tools have been designed and tested on various populations, especially adults and the elderly [4]. In the last years, however, there has been a considerable focus on children and adolescents [5]. This is a major advancement, as children under six years of age are affected by dental caries, traumatic dental injuries, malocclusion, enamel defects and dental wear [6]. Moreover, children are an important focus of dental public health research and practice [7]. However, there are as yet a limited number of measures for assessing oral health-related quality of life (OHRQoL) in children [5].

The Early Childhood Oral Health Impact Scale (ECOHIS) was developed in the United States of America by Pahel et al. [5] to assess the negative impact of oral disorders on quality of life among preschool children (0 to 5 years of age). It is structurally composed of 13 items distributed between two sections: the Child Impact Section (CIS) and Family Impact Section (FIS). The CIS has four subscales: child symptom, child function, child psychology and child self-image and social interaction. The FIS has two subscales: parental distress and family function. The scale has five rating response options to record how often an event has occurred in the life of the child: 0 = never; 1 = hardly ever; 2 = occasionally; 3 = often; 4 = very often; 5 = don't know. ECOHIS scores are calculated as a simple sum of the response codes for the CIS and FIS after recoding Don't Know responses as "missing". CIS and FIS ECOHIS scores range from 0 to 36 and 0 to 16, respectively, for which higher scores indicate a greater oral health impact and poorer OHRQoL.

### Development of the Brazilian Version of the Early Childhood Oral Health Impact Scale (B-ECOHIS)

The Brazilian version of the ECOHIS (B-ECOHIS) was obtained after the assessment of its conceptual, item and semantic equivalences [8,9] by a review committee comprised of five bilingual pediatric dentists and an expert on the cross-cultural adaptation of health-related quality of life instruments. The methodology used for the assessment of semantic equivalence involved six steps: 1- translation of the ECOHIS into Portuguese, which was performed by two translators whose first language was Portuguese; 2- a pre-test, in which the two translations were tested on a group of 20 caregivers of children aged two to five years; 3- the unification of the two versions by the review committee; 4- two back translations performed independently by two translators whose first language was English; 5- review of translations and back-translations; and 6- production of a final version of the questionnaire. Thus, the final Brazilian version of the ECOHIS resulted from a careful evaluation by experts of two translations of the original American questionnaire and back translations, but also incorporated suggestions from the target population. A detailed description of the entire process is reported elsewhere [8,9].

Concerning conceptual and item equivalence, the committee concluded that the subscales and items employed in the source instrument were relevant and appropriate to the concept of oral-health related quality of life (OHRQoL) in the Brazilian population, but considered it important to draft a new item for the child section of the questionnaire to replace the item on "missing preschool, daycare or school" because many young children in Brazil do not attend school or daycare. The new item asked if the child had "had difficulty doing daily activities (e.g., playing, jumping, running and going to school, preschool or daycare)". After consulting with the authors of the original ECOHIS, it was decided that the psychometric properties of the Brazilian version of the questionnaire should be tested with and without item replacement.

The purpose of the present study was to assess the psychometric properties (reliability and validity) of the Brazilian version of the Early Childhood Oral Health Impact Scale (B-ECOHIS).

## Methods

This study was performed in two distinct phases: a preliminary study and a field study. Each phase was developed in a different state in southeastern Brazil.

### Preliminary study

A cross-sectional study was carried out in the city of Petropolis, RJ, Brazil, in July 2005 to test the psychometric properties of the two Brazilian versions of the ECOHIS produced during the phase of semantic equivalence assessment [9]. The objective was to establish whether the Brazilian ECOHIS should include the new item suggested by the review committee or should retain the original item of the American ECOHIS, based on the evaluation of the psychometric properties of the measure. The study population comprised 150 children two to five years of age recruited at the Pediatrics Department of a public hospital and their caregivers. One previously trained researcher performed the oral examinations of the children and interviewed their caregivers. Besides the ECOHIS items, the questionnaire used in the interviews included socio-demographic data, such as age, gender and economic status. The categorization of economic status was derived from the economic classification criteria drafted by the Brazilian Advertising Association, which classifies the population into five socioeconomic categories based on the educational level of the head of the household, consumer goods owned (e.g., VCRs, DVDs, color TVs) and access to household help [10]. The questionnaire also included items designed to assess the construct validity of the B-ECOHIS (i.e., overall health status and oral health status ratings). Children's oral health status as perceived by the caregivers ("Compared to children of the same age, how would you rate your child's overall/oral health status?") was assessed by means of an ordinary scale with scores ranging from one to five, such that the responses indicating good conditions and no problems achieved the lowest scores (i.e., 0 = very good, 1 = good, 2 = fair, 3 = poor and 4 = very poor).

In order to minimize the possibility of memory playing an important role in the process of choosing answers (i.e., recalling only the first or last response options presented by the interviewer), the interviewer showed cards to the respondents with the response options to the ECOHIS and the construct validity questions typed on them and read the response options aloud.

### Field study

The field study was carried out in the city of Belo Horizonte, MG, Brazil from August 2008 through July 2009 to evaluate the final version of B-ECOHIS. Subjects were recruited from a population-based survey and randomly selected from private and public preschools (n = 49). Sample distribution was proportional to the students enrolled in these two types of schools for each region of the city. To be included in the study, the children had to be five years old, regularly enrolled in preschool, with no underlying serious medical condition, no learning disabilities and accompanied by a Portuguese-speaking caregiver. A total of 1643 caregivers of children aged five years participated in the study.

Caregivers were asked to self-administer the B-ECOHIS and a fill out a form containing socio-demographic information: age, family relation, schooling, family income level (categorized based on the minimum salary in Brazil - one minimum salary is equal to US$258.33). To characterize the families with regard to socioeconomic status, the Social Vulnerability Index (SVI) was used. This index was drafted for the city of Belo Horizonte to measure the vulnerability of the population through the determination of neighbourhood infrastructure - access to work, income, sanitation services, healthcare services, education, legal assistance and public transportation [11]. Each region of the city has a social exclusion value, which is divided into five classes. For the statistical tests, this variable was dichotomized as more vulnerable and less vulnerable. Residence address was used to classify the social vulnerability of the families.

The form also included items designed to assess the construct validity of the B-ECOHIS (i.e., overall health and oral health status ratings). The questions on overall/oral health asked the caregiver, "In general, how would you rate the overall/oral health of your child?" The response options for the two questions were "very good" (0), "good" (1), "fair" (2), "poor" (3) and "very poor" (4).

### Oral examination

In both phases of this validation study, oral examinations were performed by a single dentist previously calibrated at the public institutions. Cohen's Kappa coefficients were calculated (K = 0.90 and 1.00 for dental caries and discolored anterior teeth, respectively, in the preliminary study; K = 0.90 and 0.96 for dental caries and discolored anterior teeth, respectively, in the field study). The exams were performed in a public hospital in the preliminary study and at preschool institutions in the field study. Visual inspection of the participants' teeth was carried out in the knee-to-knee position with the aid of a flashlight. The World Health Organization (WHO) criteria for the diagnosis of decayed, missing and filled teeth (dmft) were applied [12]. The presence of discolored upper anterior teeth due to caries or trauma was also recorded. Individual cross-infection protection equipment was used. Packaged and sterilized mouth mirrors (PRISMA^®^, São Paulo, SP, Brazil), WHO probes (Golgran Ind. e Com. Ltda., São Paulo, SP, Brazil) and dental gauze were used for the examination.

The study was approved by the Human Research Ethics Committee of Rio de Janeiro State University and the Human Research Ethics Committee of the Federal University of Minas Gerais. The participants' rights were protected and children's caregivers read and signed an informed consent form prior to participation in the study.

### Statistical analysis

Reliability was assessed by tests of internal consistency and stability. Test-retest reliability was determined through the calculation of the Intraclass Correlation Coefficient (ICC) for the scores on child and family sections of the B-ECOHIS. 95% confidence intervals were estimated. The degree of reliability was assessed based on the following ICC values: ≤0.40 = weak; 0.41 to 0.60 = moderate; 0.61 to 0.80 = good; and 0.81 to 1.00 = excellent [13,14]. The B-ECOHIS was filled out twice by 50 caregivers and 178 caregivers in the preliminary and field studies, respectively, with a seven-day interval between applications. The degree of homogeneity of the child (CIS) and family (FIS) sections was assessed using Cronbach's Alpha Coefficient. Cronbach's alpha is a summary statistic, which captures the extent of agreement between all possible subsets of items. Values ≥ 0.70 were considered acceptable for comparisons between groups [15].

The ability of the B-ECOHIS to detect OHRQoL outcomes was assessed by examining the association between scores derived from this measure and a number of variables designed to indicate, both objectively and subjectively, the health status of the study population.

The convergent validity of B-ECOHIS was assessed through correlation analysis between child and family B-ECOHIS scores and two subjective self-reported health measures. The underlying hypothesis was that children whose caregivers rated their overall health and oral health status as poor would score higher on the B-ECOHIS. Convergent validity was also evaluated by examining the correlation between the CIS and FIS of the B-ECOHIS. Spearman's Correlation Coefficient was employed in these analyses.

Discriminant validity of the B-ECOHIS was determined through a comparison between the B-ECOHIS scores with one or more decayed, missing and/or filled teeth (dmft) to children without any dental disease. The hypothesis was that parents of children with dental disease and/or dental treatment experience would have higher B-ECOHIS scores (indicating worse OHRQoL) than parents of children free of dental disease. The ability of the B-ECOHIS to discriminate between children with different degrees of dental caries and children with discolored upper anterior teeth was analyzed. Among children with dental caries experience, those with more severe dental disease were presumed to obtain higher B-ECOHIS scores than children without dental caries and those with less severe dental disease. Likewise, children with discolored upper anterior teeth were presumed to obtain higher B-ECOHIS scores than children without discolored upper anterior teeth. The Mann-Whitney and Kruskall-Wallis tests were used for the analysis of these hypotheses.

Data analysis was performed using the Stata program (StataCorp LP, version 7.0, College Station, TX, USA) and Statistical Package for Social Sciences (SPSS for Windows, version 17.0, SPSS Inc, Chicago, IL, USA). The level of significance for all statistical tests was set at 0.05. Considering the field study analyses, subjects with ≥2 and ≥1 "Don't Know" responses on the child impact (n = 44) and family impact sections (n = 22), respectively, were excluded. It was possible for a respondent to be included in the analytical sample for one but not the other section of the B-ECOHIS.

## Results

### Characterization of the two samples - descriptive analysis

The preliminary study involved 150 families. The mean age of the caregivers was 31.3 years (SD = 8.7). The mean age of the children was 4.1 years (SD = 1.2); 51.3% were boys and 48.7% were girls. Most the informants were the children's mothers (87.3%) and 85.7% of the subjects were from economic classes D and C (monthly family income ≤ 517 US dollars). Fifty percent (n = 75) of the children did not attend school, preschool or daycare. Regarding the clinical status of the children, 54% had dmft = 0. The mean dmft score in this phase of the study was 2.1 (SD = 3.1) and, as expected, the severity of dental caries increased with age (i.e., dmft = 0.7, 1.2, 2.1, and 3.8 at the ages of 2, 3, 4 and 5 years, respectively). About 66.6% and 82.0% of the parents perceived their child's oral health and overall health as "very good" or "good".

The field study involved a sample totaling 1643 children's caregivers who fulfilled the inclusion criteria. The mean age of the caregivers was 33.5 years (SD = 7.6) and the mean age of the children was 65.4 months (SD = 3.7). Mothers were most often the proxy respondents (86.1%). Most of the families (71.1%) belonged to the less privileged economic levels (monthly family income ≤ 1292 US dollars), but the proportion of less and more socially vulnerable families was equal in the sample. The mean dmft of the children in this phase of the study was 1.9 (SD = 3.0). Table 1 displays the clinical disease status of the children. More caregivers rated their child's oral health as "very good" or "good" than their overall health (93.3% vs. 69.8%).

**Table 1:** Descriptive analyses: demographic and clinical characteristics of the sample - field study (n = 1643)

Demographic characteristics	Frequency	
	n	%
***Parents/Caregivers characteristics***		
**Age (years)**		
18 - 33	860	52.3
34 - 71	783	47.7
**Relationship to children**		
Mother	1414	86.1
Father	180	10.9
Other (brother/sister, grandmother/grandfather, aunt/uncle, caregiver)	49	3.0
**Level of schooling**		
> 8 years	1070	65.1
≤ 8 years	573	34.9
**Social Vulnerability Index (SVI) (residence)**		
Less vulnerable (classes III, IV, V)	900	54.8
More vulnerable (classes I, II)	743	45.2
**Family income level**		
> 5 times the minimum salary	475	28.9
≤ 5 times the minimum salary	1168	71.1
Child characteristics*		
**Gender**		
Boys	843	51.3
Girls	800	48.7
**Child's position**		
Only child	485	29.5
Youngest child	694	42.2
Other	464	28.3
**Type of preschool**		
Private	521	31.7
Public	1122	68.3
***Child's clinical disease status***		
**Decayed, missing and filled teeth**		
None	879	53.5
One or more	764	46.5
**Decayed teeth**		
None	879	53.5
1-3	409	24.9
≥ 4	355	21.6
**Discolored upper anterior teeth**		
None	1364	83.0
One or more	219	17.0

*Child age (months): mean = 65.4, SD = 3.7; range = 11

In both phases of the study, 100% of the caregivers answered the questionnaires. Impacts were more prevalent on the child section of the questionnaire (43.3% preliminary study, 35.5% field study) than the family section (35.3% preliminary study, 31.1% field study) (results not shown).

Tables 2 and 3 display the distribution of responses to the ECOHIS in the two samples. No missing responses to items were found in either phase. Regarding the preliminary study (n = 150), "felt guilty" (27.3%) was the most frequently reported impact, followed by "pain in the teeth, mouth or jaws" (26.7%) "been irritable" (17.4%), "been upset" (17.3%) and "had difficulty eating" (15.3%). The score distributions were skewed, with 56.7% and 64.7% of participants reporting "never" on all items of the child and family sections, respectively. One interviewee gave a "don't know" response to one item (i.e., "difficulty pronouncing any words") and, for the purpose of statistical analysis, this response was coded zero (never), since the response she gave to all other 12 items on the questionnaire was "never".

**Table 2:** B-ECOHIS responses of children's parents/caregivers in preliminary study (n = 150)

Impacts	Never		Hardly ever		Occasionally		Often		Very often		Don't Know		Mean(SD)
	n	%	n	%	n	%	n	%	n	%	n	%	
*Child impacts*													
How often has your child had pain in the teeth, mouth or jaws	81	54.0	29	19.3	34	22.7	5	3.3	1	0.7	0	-	0.77 (0.96)
How often has your child ....because of dental problems or dental treatments?													
had difficulty drinking hot or cold beverages	134	89.4	8	5.3	6	4.0	0	-	2	1.3	0	-	0.19 (0.63)
had difficulty eating some foods	114	76.0	13	8.7	20	13.3	3	2.0	0	-	0	-	0.41 (0.79)
had difficulty pronouncing any words	147	97.9	1	0.7	1	0.7	0	-	0	-	1	0.7	0.02 (0.18)
Missed preschool, daycare or school	131	87.3	8	5.3	10	6.7	1	0.7	0	-	0	-	0.20 (0.58)
had trouble sleeping	127	84.6	10	6.7	12	8.0	1	0.7	0	-	0	-	0.25 (0.62)
been irritable or frustrated	100	66.6	24	16.0	22	14.7	4	2.7	0	-	0	-	0.53 (0.84)
avoided smiling or laughing	137	91.4	3	2.0	8	5.3	2	1.3	0	-	0	-	0.17 (0.57)
avoided talking	145	96.6	4	2.7	1	0.7	0	-	0	-	0	-	0.04 (0.23)
daily activities (extra item)	134	89.3	4	2.7	11	7.3	1	0.7	0	-	0	-	0.19 (0.59)
*Family impacts*													
How often have you or another family member......because of your child's dental problems or treatments?													
been upset	115	76.7	9	6.0	17	11.3	5	3.3	4	2.7	0	-	0.49 (1.00)
felt guilty	101	67.2	8	5.3	29	19.3	6	4.0	6	4.0	0	-	0.72 (1.15)
taken time off from work	134	89.3	9	6.0	6	4.0	1	0.7	0	-	0	-	0.16 (0.51)
How often has your child had dental problems or dental treatments that had a financial impact on your family?	143	95.3	4	2.7	2	1.3	1	0.7	0	-	0	-	0.07 (0.37)

**Table 3:** B-ECOHIS responses of parents/caregivers of 5-year-olds in field study (n = 1643)

Impacts	Never		Hardly ever		Occasionally		Often		Very often		Don't Know		Mean(SD)
	n	%	n	%	n	%	n	%	n	%	n	%	
*Child impacts*													
How often has your child had pain in the teeth, mouth or jaws	1056	64.3	177	10.8	298	18.1	39	2.4	18	1.1	55	3.3	0.58 (0.94)
How often has your child ....because of dental problems or dental treatments?													
had difficulty drinking hot or cold beverages	1277	77.7	88	5.4	201	12.2	23	1.4	12	0.7	42	2.6	0.38 (0.81)
had difficulty eating some foods	1251	76.1	88	5.4	204	12.4	41	2.5	26	1.6	33	2.0	0.45 (0.92)
had difficulty pronouncing any words	1402	85.3	41	2.5	93	5.7	26	1.6	19	1.2	62	3.8	0.24 (0.74)
missed preschool, daycare or school	1440	87.6	58	3.5	121	7.4	10	0.6	10	0.6	4	0.2	0.23 (0.66)
had trouble sleeping	1437	87.5	42	2.6	124	7.5	22	1.3	13	0.8	5	0.3	0.25 (0.72)
been irritable or frustrated	1334	81.2	76	4.6	175	10.7	32	1.9	11	0.7	15	0.9	0.35 (0.80)
avoided smiling or laughing	1511	92.0	27	1.6	63	3.8	17	1.0	12	0.7	13	0.8	0.15 (0.60)
avoided talking	1535	93.4	30	1.8	47	2.9	12	0.7	5	0.3	14	0.9	0.11 (0.49)
*Family impacts*													
How often have you or another family member......because of your child's dental problems or treatments?													
been upset	1269	77.2	46	2.8	212	12.9	61	3.7	52	3.2	3	0.2	0.52 (1.05)
felt guilty	1237	75.3	37	2.3	239	14.5	63	3.8	61	3.7	6	0.4	0.58 (1.10)
taken time off from work	1434	87.3	38	2.3	144	8.8	18	1.1	6	0.4	3	0.2	0.25 (0.69)
How often has your child had dental problems or dental treatments that had a financial impact on your family?	1438	87.5	56	3.4	94	5.7	30	1.8	15	0.9	10	0.6	0.24 (0.72)

In the field study (n = 1643), the most frequently reported items by the parents/caregivers on the child impact section were "pain" (21.6%), "difficulty drinking" (14.3%), "difficulty eating" (16.5%) and "irritation" (13.3%). Regarding the family impact section, the most frequent items were "been upset" (19.8%) and "felt guilty" (22.0%). As observed in the preliminary study, 51.5% and 64.6% of the participants reported "never" on all items of the child and family sections, respectively. About 11.9% of participants responded "Don't Know" to one or more questions. Caregivers answered "Don't Know" most often for items related to "pain" and "difficulty drinking, eating and pronouncing words" on the child impact section. About 9.0%, 2.2% and 0.7% answered "Don't Know" to one, two and three or more questions, respectively. B-ECOHIS scores ranged from 0 to 19 on the child section (Mean = 2.6; SD = 3.3), and 0 to 13 on the family section (Mean = 1.4; SD = 2.2) in the preliminary phase. In the field study, B-ECOHIS scores ranged from 0 to 34 on the child section (Mean = 2.7; SD = 4.5) and 0 to 16 on the family section (Mean = 1.6; SD = 2.7).

### Reliability

Internal consistency was assessed for each of the two sections using Cronbach's alpha coefficient. In the preliminary study, Cronbach's alpha was 0.74 for the child section and 0.59 for the family section. Replacing the original item "missing school, preschool or daycare" with the item "difficulty doing daily activities" in the child section of the questionnaire did not change the internal consistency of the scale or subscale. Reproducibility of the sections scores was assessed using the ICC (child section: 0.98; family section: 0.97). The replacement of one item in the child section of the questionnaire did not affect the test-retest reliability. In the field study, Cronbach's alpha coefficient for internal consistency of items on the child and family sections was 0.80 and 0.76, respectively. The ICC for the test-retest reliability was 0.99 for the child and family impact sections.

### Construct Validity

Construct validity was measured using Spearman's Correlation Coefficient between the scores obtained on the two sections of the B-ECOHS and 1- overall health status rating, 2- oral health status rating, and 3- the child and family sections of the B-ECOHIS. In both phases of this study, data with respect to perceived oral health status were skewed in a favorable direction (i.e., higher frequency of "good" and "very good" responses, indicating a positive perception of oral health). Despite being statistically significant, correlations were weak on the overall health rating (Table 4). In the preliminary study, the replacement of one item in the child section of the questionnaire did not affect the correlation coefficients. The correlation between the child and the family sections of the measure was statistically significant (Preliminary study: r_s _= 0.54, p ≤ 0.001; Field study: r_s _= 0.62, p ≤ 0.001). Children whose caregivers rated their overall health and oral health status as poor would score higher on the B-ECOHIS.

**Table 4:** B-ECOHIS findings for convergent validity - preliminary and field studies

Variable	Child Section Spearman's r	Family Section Spearman's r
***Preliminary study***		
Overall health status rating	0.21*	0.22*
Oral health status rating	0.51**	0.58**
***Field study***		
Overall health status rating	0.13**	0.09**
Oral health status rating	0.45**	0.47**

Statistically significant at *p ≤ 0.01, **p ≤ 0.001(2-tailed)

Discriminant validity of the B-ECOHIS was determined through the following comparisons: scores for children with one or more decayed and/or treated teeth to those without any dental disease; scores between children with different degrees of dental caries; and scores of children with discolored upper anterior teeth to those without discolored upper anterior teeth. The groups with known clinical conditions were differentiated from those without such conditions on the two sections of the scale (p ≤ 0.001) (Table 5). It is interesting to note that, in the preliminary study, there was no difference on the child impact section between children who exhibited 1 to 3 and ≥4 decayed teeth (p > 0.05). This may be explained by the proximity of the mean and median B-ECOHIS scores for both groups. In the preliminary study, these results did not change after the item "missing school, preschool or daycare" was replaced with "difficulty doing daily activities".

**Table 5:** Findings for discriminant validity in preliminary and field studies

Variables	Child Impact section				Family Impact section			
		
		Mean B-ECOHIS Score (SD)				Mean B-ECOHIS Score (SD)		*P-*value, Kruskall-Wallis test
								
	n	[Median B-ECOHIS Score]	Difference	*P *value Kruskall-Wallis test	n	[Median B-ECOHIS Score]	Difference	
***Preliminary study***								
**Decayed, missing and/or filled teeth**								
None _(a)_	81	1.2 (2.1)			81	0.4 (1.1)		
		[0.0]	a,b***	-		[0.0]	a,b***	-
One or more _(b)_	69	4.2 (3.7)			69	2.7 (2.5)		
		[4.0]				[2.0]		
**Decayed teeth**								
None _(a)_	90	1.3 (2.1)			90	0.5 (1.2)		
		[0.0]				[0.0]		
1-3 _(b)_	36	4.1 (4.0)	a,b***;	p < 0.001	36	2.3 (2.3)	a,b***;	p < 0.001
		[3.0]	a,c***;			[2.0]	a,c***;	
≥4 _(c)_	24	5.2 (3.4)	b, c^n.s^		24	3.7 (2.7)	b,c*	
		[5.5]				[2.0]		
**Discolored upper anterior teeth**								
None _(a)_	132	2.2 (3.1)			132	1.1 (1.9)		
		[1.0]	a,b***	-		[0.0]	a,b***	-
One or more _(b)_	18	5.2 (3.5)			18	3.7 (3.1)		
		[5.5]				[3.0]		
***Field study***								
**Decayed, missing and/or filled teeth**								
None _(a)_	862	1.2 (2.6)			875	0.6 (1.6)		
		[0.0]	a,b***	-		[0.0]	a,b***	-
One or more _(b)_	737	4.5 (5.6)			746	2.7 (3.3)		
		[2.0]				[2.0]		
**Decayed teeth**								
None _(a)_	980	1.4(2.8)			997	0.8 (1.8)		
		[0.0]				[0.0]		
1-3 _(b)_	387	3.2 (4.4)	a,b***;	p < 0.001	389	2.1 (3.0)	a,b***;	p < 0.001
		[1.0]	a,c***;			[0.0]	a,c***;	
≥4 _(c)_	232	7.3 (7.0)	b,c***		235	4.2 (3.7)	b,c***	
		[5.5]				[4.0]		
**Discolored upper anterior teeth**								
None _(a)_	1364	2.5 (4.4)			1381	1.5 (2.7)		
		[0.0]	a,b***	-		[0.0]	a,b***	-
One or more _(b)_	219	3.0 (4.1)			220	2.0 (2.9)		
		[1.0]				[0.0]		

Statistically significant at *p < 0.05, **p ≤ 0.01, ***p ≤ 0.001, based on Mann-Whitney Test; n.s. - Statistically non-significant based on Mann-Whitney Test

## Discussion

OHRQoL measures have been the target of investigations in the oral healthcare field and have proven valuable in assessing oral health needs, especially among the adult population [16]. In recent years, OHRQoL measures have been designed for the child population [17]. However, these instruments are not yet available in all countries or languages. Most questionnaires have been drafted in English-speaking countries and adapted for use in other countries [18]. The translation and the testing of psychometric properties are important steps to ensuring the quality of a cross-cultural adaptation of an OHRQoL measure [19]. Considering the differences between social, cultural and economic aspects, the availability of cross-culturally valid, multi-lingual versions of instruments is important to obtaining reliable, comparable data [20].

The present study determined the reliability and validity of the ECOHIS, which is a multidimensional assessment tool for measuring the negative impact of oral problems on quality of life among preschool children (0-5 years of age) that has been cross-culturally adapted to Brazilian Portuguese [9]. In the first phase of the present study, both versions of B-ECOHIS (a version including the original item "missing preschool, daycare or school" and another replacing this item with "had difficulty doing daily activities") were tested with parents/caregivers of children who attended school and those who did not attend school. No differences in psychometric properties between versions were found. It is likely that the responses of the parents/caregivers of children who did not attend school/daycare for the item 'missed preschool, daycare or school' was 'never', which did not interfere in the psychometric analysis of the instrument. Therefore, the B-ECOHIS was maintained as originally drafted by Pahel et al. [5].

Assessment instruments should be reproducible over time, that is, they should produce similar results on two or more administrations to the same individual, provided that the general clinical state has not been altered. The analysis of test-retest reliability suggests the adequate stability of the instrument. The seven-day interval between interviews was important to diminishing the probability of systemic alterations in the clinical condition of the patient. It is recommended that the interval between measurements be long enough to reduce the effects of memory and short enough to diminish the likelihood of systemic alterations. Although the definition of this interval is arbitrary, a period of two to 14 days is considered adequate [21-23]. The B-ECOHIS was answered twice and demonstrated good stability during the preliminary and the field studies.

Internal consistency was calculated using Cronbach's Alpha Coefficient for the child and family impact sections and demonstrated adequate homogeneity (α ≥ 0.70) in the field study. As in the original version (α = 0.91 for CIS; α = 0.95 for FIS) [5], French version (α = 0.79 for CIS and FIS) [17], Chinese version (α = 0.87 for CIS; α = 0.85 for FIS) [19] and Farsi version (α = 0.89 for CIS; α = 0.85 for FIS) [24], the final version of B-ECOHIS performed reliably. One point that shoud be addressed regards the values of Cronbach's alpha in the preliminary study: 0.74 for the child section and 0.59 for the family section. Cronbach's alpha for the family section was low. Aspects such as the number of items on this section and sample size could have influenced this result. In the field study however, Cronbach's alpha for the family section was 0.76.

Correlations were obtained between the scores of the instrument and global measures of overall and oral health as well as on the child and family sections of the B-ECOHIS. The same was found with the original version of the ECOHIS [5]. Measures concerning quality of life components should reflect the values of the subjects. However, lay people are sometimes asked to fill out questionnaires that do not reflect their real concerns, but rather the values of physicians, social scientists or other experts [25]. We believe that items on the questionnaire concerning aspects of daily life could often reveal experiences that are relatively less important to the target population [25]. This may explain the weak correlations found for convergent validity. In the dental field, it is common for lay people to consider the mouth as a focus unlinked to the individual as a whole, which could influence the comprehension of the relationship between oral health and quality of life.

Discriminant validity analysis is considered a useful method in the differentiation of groups that are known to be distinct [5,18]. In the present study, the occurrence of oral problems implied limitations and difficulties, confirming the hypothesis formulated with regard to the construct validity of the B-ECOHIS: 1- individuals with one or more decayed, missing and/or filled teeth (dmft) obtained higher scores on the B-ECOHIS (indicating worse OHRQoL) than children without dental disease; 2- parents of children with dental disease and/or dental treatment experience obtained higher B-ECOHIS scores than those of children free of dental disease experience; 3- among children with dental caries experience, those with more severe dental disease obtained higher B-ECOHIS scores than those without dental caries and those with less severe dental disease; 4- children with discolored upper anterior teeth obtained higher B-ECOHIS scores than those without discolored upper anterior teeth.

Analyzing the distribution of items in both phases of the study, the most frequently reported items on the two sections of the scale were practically the same as those reported in previous validity studies carried out in Quebec, Canada [17] and Hong Kong, China [19]. On the child impact section, the most prevalent items were related to "pain", "eating" and "irritation". On the family impact section, the most prevalent item was "been upset".

As done in the original version, the number and distribution of "I don't know" responses were taken into account. In the field study, 11.9% of subjects answered "I don't know" to one or more questions, which is somewhat higher than the 7% reported for the original ECOHIS [5], but similar to that found in the French ECOHIS [17]. An "I don't know" response option is essential in studies that assess the participants' perceptions of health or quality of life of another individual, as it reflects a particular characteristic of the phenomenon under evaluation [7]. Considering the management of this response option, Jokovic et al. [7] proposes the following: 1- exclude subjects with such responses; 2- use adjusted scores; or 3- drop items from the questionnaire that have high proportion of "I don't know" responses. As the present study was conducted on a large sample (population-based survey) and the proportion of such responses was low, the decision was made to exclude subjects with ≥ 2 and ≥ 1 "I don't Know" responses on the child impact and family impact sections, respectively (2.7% and 1.3% of total sample). Jokovic and colleagues [7] found that excluding subjects or using adjusted scores did not affect the validity analyses.

The present study has particular characteristics that should be recognized. In the preliminary and field studies most of the families were from less privileged economic classes. It should be pointed out that parents' perceptions regarding their child's oral health could be influenced by socioeconomic conditions [26]. A recent Brazilian study confirmed that parents who earned a lower income were more likely to rate their child's oral health as 'poor' [27]. We believe that this point does not affect the results of this validation study, as the prevalence of the negative impact was not high. Both studies were performed with different sample populations. The first was developed with a convenience sample, like the studies carried out in the United States of America, Canada, China and Iran [5,17,19,24], whereas the field study was nested in an epidemiological survey. A large percentage of validation studies carried out in the field of dentistry use convenience samples. However, it is recognized that a large sample size leads to more accurate parameter estimates, which leads to a greater ability to meet the aims of the study [28]. Thus, the decision was made in the present investigation to employ a population-based study with a large sample size in order to produce more accurate results. This strategy has been used in other fields of knowledge, such as medicine. The age of the children, place of recruitment and mode of administration of the B-ECOHIS were also different. Regarding the self-administration versus interviewer-administration of a measure, a number of studies have demonstrated that the mode of administration does not affect the performance of the measure [29,30]. Moreover, the measure was administered in two different states in southeastern Brazil. Despite the methodological differences, the psychometric properties of the B-ECOHIS proved similar in both phases. This offers further evidence of the validity and reliability of the measure. However, it should be pointed out that, due to the cultural diversity of Brazil, using the B-ECOHIS in other regions may require some adaptation and such cases should involve further psychometric testing.

## Conclusion

The Brazilian version of the Early Childhood Oral Health Impact Scale (B-ECOHIS) exhibited adequate properties regarding reliability and construct validity. This suggests its usefulness as a parameter in studies assessing the impact of oral disorders on the quality of life of young children and their families. Decisions for the implementation of improvements to oral healthcare services may be adopted based on the impact of oral disorders and their effect on quality of life.

## Abbreviations

B-ECOHIS: Brazilian version of Early Childhood Oral Health Impact Scale; CIS: Child impact section; dmft Decayed, missing and/or filled teeth; DVD: Digital video disc; ECOHIS: Early Childhood Oral Health Impact Scale; F: Field study; FIS: Family impact section; ICC: Intraclass Correlation Coefficient; MG: Minas Gerais; OHRQoL: Oral health-related quality of life; P: Preliminary study; RJ: Rio de Janeiro; SVI: Social Vulnerability Índex; TV: Television; VCR: Video cassette recorder; WHO: World Health Organization

## Competing interests

The authors declare that they have no competing interests.

## Authors' contributions

ACS, BHO, FCT, ATL, IAP and SMP conceptualized the rationale and design of the study. ACS, BHO and ATL contributed to the statistical analysis and interpretation of the data. ACS, BHO, ATL and SMP drafted the manuscript. All authors read and approved the final manuscript.

## Pre-publication history

The pre-publication history for this paper can be accessed here:

http://www.biomedcentral.com/1472-6831/11/19/prepub
